# Supplementary material for: Mechanistic home range capture–recapture models for the estimation of population density and landscape connectivity
Source: Ecology. 2025 Feb 24;106(2):e70046. doi: 10.1002/ecy.70046 (PMC11848510; doi:10.1002/ecy.70046)
Supplement: Supplementary file 1 — Appendix S1: [file ECY-106-e70046-s002.pdf]

Supporting Information. Mechanistic home range capture–recapture models for the estimation of population density and landscape connectivity. Keita Fukasawa and Daishi Higashide. Ecology.

Appendix S1 Technical details in the finite difference method.

We applied a finite difference method to derive a numerical solution to an advection–diffusion model of utilization distribution in a discretized space. In this study, a finite difference method was applied in dynamic setting for the animal movement simulation as well as static setting for estimation of ADCR.

Discretization schemes for a finite difference method have trade-offs between precision and numerical stability (Hirsch 2007). To ensure numerical stability for a diverse range of parameter values, we applied a first-order upwind scheme for advection and second-order central difference scheme for diffusion. Discretizing time by backward differentiation, eqn. 2 is approximated by a transition matrix for a cell-wise probability vector $\mathbf{p} = (p_1, p_2, \ldots, p_N)$ is obtained as follows:

$$\mathbf{p}_{t+\Delta t} = \left\{\mathbf{I} + \left(-c\left(\frac{\mathbf{A}_{x1}\mathrm{diag}(|\mu_x - \mathbf{x}|)}{\Delta x} + \frac{\mathbf{A}_{y1}\mathrm{diag}(|\mu_y - \mathbf{y}|)}{\Delta y}\right) + \mathrm{diag}(\mathbf{d}^{1/2})\left(\frac{\mathbf{A}_{x2}}{\Delta x^2} + \frac{\mathbf{A}_{y2}}{\Delta y^2}\right)\mathrm{diag}(\mathbf{d}^{1/2})\right)\Delta t\right\}\mathbf{p}_t$$

(eqn. S1)

where $\mathbf{A}_{x1}$ and $\mathbf{A}_{y1}$ are non-symmetric adjacency matrices corresponding to the advection term for the x and y axis, respectively. $\mathbf{A}_{x2}$ and $\mathbf{A}_{y2}$ are symmetric adjacency matrices corresponding to the diffusion term. Off-diagonal elements ($i, j, i \neq j$) in the adjacency matrix for advection are 1 if cell i and j are neighbors and the direction of drift is i -> j and are 0 otherwise. Off-diagonal elements in the adjacency matrix for diffusion are 1 if

cell i and j are neighbors. To satisfy the principle of probability mass conservation, diagonal elements of both types of adjacency matrices were determined so that all column sums are 0. Δx, Δy and Δt are resolutions of discretization for the x axis, y axis, and time, respectively. $\mathbf{x}$, $\mathbf{y}$, and $\mathbf{d}$ are vectors of the x and y coordinates and diffusion coefficient of grid cells, respectively. The function diag() indicates vector-to-diagonal matrix conversion. The diffusion coefficient $\mathbf{d} = (d_1, d_2, \ldots, d_N)$ is positive-valued and often assumed to be dependent on cell environmental variables (e.g., topographic and land cover variables).

Using an explicit method, $\mathbf{p}_{t+\Delta t}$ is projected from $\mathbf{p}_t$. A realization of animal movement at time $t+\Delta t$ follows categorical distribution with probability vector $\mathbf{p}_{t+\Delta t}$. This is a discrete space analogue of the step selection function defined by the advection-diffusion equation (Potts and Schlägel 2020), and this stochastic model was used to simulate animal movement.

Home range in this system is defined as an equilibrium utility distribution, p^*, which is the solution of p when $\delta p/\delta t = 0$. Discrete approximation of the utility distribution, $\mathbf{p}^*$, is given by solving the following sparse linear system:

$$\left(-\left(\frac{\mathbf{A}_{x1}\mathrm{diag}(|\mu_x-\mathbf{x}|)}{\Delta x}+\frac{\mathbf{A}_{y1}\mathrm{diag}(|\mu_y-\mathbf{y}|)}{\Delta y}\right)+\mathrm{diag}\left(\left(\frac{\mathbf{d}}{c}\right)^{\frac{1}{2}}\right)\left(\frac{\mathbf{A}_{x2}}{\Delta x^2}+\frac{\mathbf{A}_{y2}}{\Delta y^2}\right)\mathrm{diag}\left(\left(\frac{\mathbf{d}}{c}\right)^{\frac{1}{2}}\right)\right)\mathbf{p}=\mathbf{0}.$$

(eqn. S2)

Given μ_x, μ_y and $\mathbf{d}/c$, this equation has a unique solution with constraint $\mathbf{1}^T\mathbf{p} = 1$.

References

Hirsch, C. 2007. Numerical computation of internal and external flows: the fundamentals

of computational fluid dynamics. Elsevier.

Potts, J. R., and U. E. Schlägel. 2020. Parametrizing diffusion-taxis equations from animal movement trajectories using step selection analysis. Methods in Ecology and Evolution 11:1092–1105.
