# Supplementary material for: Mechanistic home range capture–recapture models for the estimation of population density and landscape connectivity
Source: Ecology. 2025 Feb 24;106(2):e70046. doi: 10.1002/ecy.70046 (PMC11848510; doi:10.1002/ecy.70046)
Supplement: Supplementary file 6 — Appendix S6: [file ECY-106-e70046-s005.pdf]

Supporting Information. Mechanistic home range capture–recapture models for the estimation of population density and landscape connectivity. Keita Fukasawa and Daishi Higashide. Ecology.

Appendix S6. Supplementary Tables.

Table S1 Target parameter ranges for the neutral landscape generator, rflsgen. The parameter names were based on Justeau-Allaire et al. (2022). Parameters not shown were set to default values.

Parameter name	Minimum	Maximum
Number of patches	5	10
Patch area	100	400
Total class area	1000	2000
Effective mesh size	0	200

Reference

Justeau-Allaire, D., G. Blanchard, T. Ibanez, X. Lorca, G. Vieilledent, and P. Birnbaum. 2022. Fragmented landscape generator (flsgen): A neutral landscape generator with control of landscape structure and fragmentation indices. Methods in Ecology and Evolution 13:1412–1420.

Table S2 Contingency table on the correctness of sign (i.e. positive or negative) of landscape effect on connectivity estimated by ADCR and SCR with the least-cost path for the simulation scenario of sparse data and biased detector alignment. The significance was determined by non-overlap of 95% confidence interval with 0.

		effect on cost by SCR with the least-cost path				
		incorrect sign (significant)	incorrect sign (not significant)	correct sign (not significant)	correct sign (significant)	Total iterations
effect on permeability by ADCR	incorrect sign (significant)	0	0	0	0	0
	incorrect sign (not significant)	0	1	1	0	2
	correct sign (not significant)	0	2	29	7	38
	correct sign (significant)	0	2	11	47	60
	Total iterations	0	5	41	54	100
